# Inhibitory control during selective retrieval may hinder subsequent analogical thinking

**DOI:** 10.1371/journal.pone.0211881

**Published:** 2019-02-12

**Authors:** Tania M. Valle, Carlos J. Gómez-Ariza, M. Teresa Bajo

**Affiliations:** 1 Research Center for Mind, Brain and Behavior, University of Granada, Granada, Spain; 2 Department of Psychology, University of Jaen, Jaen, Spain; University of Birmingham, UNITED KINGDOM

## Abstract

Analogical reasoning is a complex cognitive activity that involves access and retrieval of pre-existing knowledge in order to find a suitable solution. Prior work has shown that analogical transfer and reasoning can be influenced by unconscious activation of relevant information. Based on this idea, we report two experiments that examine whether reduced access to relevant information in memory may further disrupt analogical reasoning unwittingly. In both experiments, we use an adaptation of the retrieval practice paradigm [1] to modulate memory accessibility of potential solutions to a subsequent set of analogy problems of the type ‘A is to B as C is to ?’. Experiment 1 showed a retrieval-induced impairment in analogical problem solving. Experiment 2 replicated this finding and demonstrated that it cannot be due to the deliberative episodic retrieval of the solutions to the analogies. These findings, predictable from an inhibitory framework of memory control, provide a new focus for theories of analogical transfer and highlight the importance of unconscious memory processes that may modulate problem solving.

## Introduction

Memory plays a prominent role in our everyday reasoning activities by allowing us to access relevant past experiences, which could thus be applied to new situations [2]. In the context of problem solving, considerable attention has received the way in which we access, retrieve and use stored knowledge to solve new problems [3–7]. Much of this research has focused on inductive reasoning processes such as analogical thinking, which involves generating novel connections and transferring information from a well-known domain to a new one on the basis of similarities and correspondences [8]. Analogical reasoning is seen as a fundamental tool in a wide variety of problem-solving contexts such as scientific discovery [9], mathematics [10,11] or creative problem solving [12]. In all these contexts, memory plays a central role since information from one domain has to be accessed and applied to a different one. For instance, ‘the solar system’ analogy has been used to explain the atomic structure (how a planet orbits the sun can be thought as analogous to the way in which an electron orbits an atomic nucleus), but this analogy only can be inferred if the person already knows about and have access to the stored information regarding the structure of the solar system [13]. There is considerable support for the claim that memory accessibility is crucial in analogical thinking; namely, the relevant source idea and potential analogies must be accessed, selected and retrieved from all the related information stored in long-term memory in order to map them and generate new inferences [14].

A growing body of literature has started to examine how memory accessibility may influence analogical reasoning [3,15–18]. Since memory is a dynamic process, there are mechanisms that can either facilitate or hinder access to the information stored in long-term memory and, thus, have an indirect influence on the reasoning process. A key issue concerns the degree to which participants spontaneously access and use relevant information during analogical reasoning. Hence, in some experiments, participants are exposed to information that would be useful for a subsequent problem-solving task without being informed about the relevance of the provided information. Even though research has demonstrated that people often fail to take advantage of this information [4,5,19], a number of studies have also found evidence of how prior activated knowledge impact reasoning and problem solving [2,9,17,20–22].

However, there is some controversy on whether the possible impact of previous knowledge can influence analogical reasoning in both explicit and implicit manners. For example, it has been argued that awareness of encoding and retrieval processes is required to flexible application of knowledge in successful analogical transfer [23,24]. In this line, some studies have used explicit cues to prompt participants to remember analogous previous problems as a way of increasing transfer effects [4,5,10,19,25]. Gick and Holyoak [5], for example, had participants generate a solution to treat a tumor without using radiation that would destroy the surrounding tissue. In the control condition where no analogical source was presented, only a 10% of participants came up with the solution. When previously provided with a similar solution but applied in a different incidental context without any hint that they could use the solution to approach the target problem, about 30% of participants generated analogous solutions. Notably, when these participants were explicitly told that remembering the previous story might be helpful to produce a solution to the problem, the percentage of convergence solutions increased to 80%. These results suggest that spontaneous access to potential useful analogies may be difficult even when they are available in memory, unless explicit cues are provided.

In contrast, recent studies have shown that analogical mapping may act without an explicit prompt and without awareness of how solutions become accessible [2,9,17,20–22]. For example, in Gross and Greene's [22] study participants learned a control sequence of faces (A>B, B>C, where A>C is usually inferred) or a transverse pattern set (A>B, B>C and C>A). Then, they learned a partial set of new faces (X>Y and Y>Z) and were tested for transfer on the new pair (X?Z). The group that was exposed to the transverse pattern C>A adopted the transverse pattering relations and selected Z>X at a greater extent than the control group, which rarely chose that pattern. Importantly, analogical transfer occurred even though participants were not explicitly prompted to do so and in the absence of awareness. Moreover, unconscious analogical thinking has been observed in a variety of context such as problem solving [9,21], text comprehension [20], false memories [17] and in absence of deliberate analogical strategies [22]. In problem-solving contexts, for example, Schunn and Dunbar [9] showed that knowledge about a domain could enhance performance of a reasoning problem from a different domain through implicit priming. In their experiment, participants were first asked to solve a biochemistry problem by discovering which viruses were in a dormant state as a consequence of an inhibitory process. In a second session, participants solved an unrelated molecular genetics problem whose solution involved inhibition of a set of genes. Both biochemistry and molecular genetics problems solutions involved the same concept of inhibition. The authors found that participants that were initially exposed to the biochemistry problem were more likely to propose the concept of inhibition to solve the second problem and solved it faster than control participants. Furthermore, participants reported not to be aware of the relationship of the solutions between the two tasks. Similarly, Day and Goldstone [21] found evidence of analogical transfer of strategies between two unrelated tasks. Participants who first learned how to solve a concrete perceptual simulation of a physical system were better at solving a task with very dissimilar domain and appearance, which involved an analogous structure and strategy. In addition, the transfer was independent of the participants’ explicit reports about their awareness of the application of the analogous strategy.

Other results suggest that false memories could also prime problem solving and reasoning tasks as true memories do. Howe, Garner, Threadgold and Ball [17] primed solutions in analogical problem solving by exposing participants to Deese-Roediger-McDermott (DRM) lists. In a standard DRM experiment, participants first study a list of words that are associates (e.g., tiger, circus, tamer, roar…) of a critical semantically related item (e.g., lion), which is never presented at the study. After a retention interval, the participants’ memory for the studied words is tested. The usual result is that participants produce or endorse the critical lure as a previously studied word as a consequence of the semantic relatedness between studied words and critical lures. In Howe et al.’s [17] study, after a free recall test, participants solved analogies of the type ‘A is to B as C is to D’ in which they had to generate the ‘D’ term (e.g., peace is to dove as courage is to ?). Some of the analogies’ solutions were critical lures of the DRM lists (false memory primed solutions, e.g., lion), whereas the remaining solutions were neither included in the lists nor related to them (unprimed solutions). Results revealed that participants solved significantly more analogies whose solutions were primed by false memories (critical lures) than analogies whose solutions were not primed. When participants were questioned on whether they noticed the two tasks were related, most reported that they did not think that there was a connection between the two phases. Taken together, these results seem to suggest that prior activation of knowledge by an unrelated task may make relevant information more readily accessible for solving analogical problems. Thus, presenting certain pieces of information has the potential to prime access to related information and implicitly enhance analogical thinking.

The question here, however, is whether situations that temporarily render relevant memories inaccessible might, in turn, hinder analogical problem solving. Given that the generation of potential solutions relies on access to memory, if potential solutions are made less accessible and harder to retrieve during problem solving, performance should be impaired. Hence, any process that reduces the accessibility of relevant information in memory might hamper analogical reasoning. A control mechanism that is thought to decrease activation of memory representations is inhibition, which would be in charge of downregulating irrelevant but competing memories to facilitate access to relevant ones [1]. The role of inhibitory control as a mechanism to overcome interference during episodic retrieval has been extensively studied with the retrieval practice (RP) procedure. In this procedure, participants engage in practicing retrieval of only some of the previously studied items. While this selective retrieval usually leads to better accessibility (enhanced recall/recognition) of practiced items, it also causes the temporary inaccessibility (worse recall/recognition as compared to control items) of related non-practiced items that compete for retrieval during practice. According to an inhibitory framework (i.e., [26–30]), this retrieval-induced forgetting (RIF) phenomenon is the aftereffect of inhibitory control exerted during selective retrieval, so that competing information that was previously inhibited remains in a below-baseline activation state that renders it less accessible if, later, this information becomes relevant and has to be retrieved. While most research on RIF has been conducted by using recall and recognition tasks to look into the consequences of inhibitory control (for reviews, see [31,32]), the retrieval practice (RP) procedure has also proved to be an useful tool to study the influence of memory activation and inhibition on thinking and decision making.

For example, Iglesias-Parro and Gómez-Ariza [33] found that participants’ judgments about the suitability of imaginary prospective candidates for employment could be biased by means of reduced access to relevant information. In their study, they used an adapted version of the retrieval practice paradigm [1] so that participants were first presented candidates for a telephone insurance seller job position who were described with relevant (i.e. nice voice or verbal fluency) and irrelevant (i.e. tall or single) attributes. Then, participants practiced retrieval of irrelevant attributes related to one of the candidates. Lastly, participants were asked to choose the best candidate for the job position before a final memory test. As expected, participants selected the candidate whose irrelevant attributes were not selectively retrieved, so showing selective forgetting of the competing applicant’s relevant attributes. Hence, making a decision in the context of a personnel selection task was biased by means of the retrieval-induced inhibition of job applicants’ traits. Consistent with this finding, one recent study also demonstrated that creative thinking might be adversely affected by reduced accessibility to relevant information [34].

Taken together, these findings suggest that reduced access to relevant representations in memory would result in poor performance in any problem-solving task as long as it strongly relies on memory accessibility. With the aim of putting to an empirical test this idea, the present experiments focus on analogical reasoning. The idea is that if potential solutions become less accessible from memory as an aftereffect of selective retrieval (i.e., via inhibitory control), subsequent performance on analogical problems should be impaired. Of special relevance here, given the controversy regarding whether modulating accessibility of previous knowledge can implicitly influence analogical reasoning [23,24], we designed the present experiments so that if any, the possible negative effect of selective retrieval on analogical reasoning did not rely on explicit retrieval of previously presented information. Thus, we did our best to avoid that participants noticed the connection between the retrieval practice and the problem solving phases.

## Experiment 1

Experiment 1 aimed to determine whether memory accessibility might unconsciously impact analogical problem solving. Specifically, we were interested in exploring whether items that had previously been the target of inhibitory control during selective retrieval were less likely to be chosen as solutions in an analogical reasoning task. To this end, the RP paradigm [1] was adapted to manipulate the accessibility of candidate words as solutions for subsequent analogy problems. In the standard RP paradigm, participants typically study a list of category-exemplar pairs (e.g., Fruit-Banana, Fruit-Melon, Furniture-Shelving, Furniture-Wardrobe). Then, they are asked to selectively retrieve half of the items of half of the categories by a given a cue (e.g., Fruit-Ba____). Finally, a recall (or recognition) test is administered for all the studied items. As previously mentioned, selective retrieval usually facilitates later recall of practiced (Banana) items compared to control items (unrelated and unpracticed; Shelving and Wardrobe). On the contrary, unpracticed related items (Melon) are worse recalled than control items, which may be understood as an aftereffect of inhibitory control that acted on these competing items in memory during the selective retrieval of practiced items (e.g., [26,32,35]).

With the idea of exploring if memory inhibition may also affect analogical thinking, in the present experiments we replaced the final memory test typically used in the RP procedure with a set of analogical problems whose solutions matched some of the studied words. The analogical problems consisted of four-term analogies of the type ‘A is to B as C is to D’ generally employed in standardized intelligence and vocabulary knowledge tests [36]. In this type of analogies, the A, B, and C terms are presented and solvers must find the D term to complete the sentence. That is, the participant had to be able to connect the different terms by finding a relationship between the two first pair of concepts in order to map it to the third word and find a suitable solution (e.g., BIRD is to FEATHERS as DOG is to ?). We predicted that to the extent that selective retrieval leads to the inhibition of competing items in memory, if these competing items turn out to be potential solutions in a subsequent test of analogical reasoning the inhibited words should be less accessible and harder to produce as D terms of analogy problems. This expectation only follows if analogical reasoning makes use of previously activated/inhibited knowledge in an implicit manner.

### Method

#### Participants

30 undergraduate students (mean age = 19.67 years; SD = 1.92) from the University of Granada participated in the experiment in exchange for course credit. This sample size was determined on the basis of the number of participants included in related previous studies that looked into the effects of selective retrieval on problem solving (e.g., [33,37]). All participants were native Spanish speaker, had normal or corrected-to-normal vision and gave their written consent to participate in the experiment by signing the appropriate informed consent paperwork. The Ethics Committee of the University of Granada approved the procedure of this study.

#### Materials

We used the items employed by Bajo, Gómez-Ariza, Fernandez and Marful ([38]; see also [29,33]) with some modifications and the addition of new categories and items. The material consisted of fifty-four Spanish words from nine different orthography-based categories. Two additional categories of two words each were created and used as fillers at the beginning and at the end of the study lists in order to control for primacy and recency effects. Each orthographic category was composed of six (semantically unrelated) words that shared their first two letters (e.g., Maquillaje, Marinero, Matanza, Madurez, Maleta and Manual for the category MA). All the words were chosen according to their lexical frequency from the [39] database. Each category was composed of three medium-high frequency words (range = 34–98, M = 58.78) and three medium-low frequency words (range = 10–36, M = 20.15). Medium-low lexical frequency words were used as to-be-practiced (Rp+), unpracticed control (Nrp+) and unprimed (Up+) items depending on the across-participants counterbalance condition. Medium-high lexical frequency words were used as related unpracticed (competing) items (Rp-), unpracticed control (Nrp-) items, or unprimed words (Up-) also depending upon the counterbalance version. As in previous RIF studies, the idea was to have competitive enough (high-frequency) Rp- items to maximize the need of inhibitory control during the phase of selective retrieval of the (low-frequency) Rp+ items ([26]; See also [38]). Moreover, the words selected (a) did not share apparent semantic relationship among the words belonging to the same category (b) were between two and five syllable lengths and (c) had a unique third letter. Six counterbalanced versions of the study material were created and used across participants so that every category rotated and appeared in the practiced, unpracticed, and non-studied conditions. In each version, three categories were studied and practiced (i.e., BA, DE, MA) and produced Rp+ and Rp- items; three categories were studied but not practiced and produced Nrp+ and Nrp- control items (i.e., CA, PE, FA) and the last three categories were unstudied and produced Up+ and Up- unprimed items (i.e., DI, RE, TA).

Fifty-four analogical reasoning problems of the logical type A:B::C:D (A is to B as C is to ?) used in standardized tests were created (e.g., Miller Analogies Test (MAT) or Scholastic Aptitude Test (SAT); [40,41]). Each problem could be solved with one of the fifty-four words from the nine categories described above (AVARICIA es a GENEROSIDAD como INFANTILISMO a … whose solution would be MADUREZ; GREED is to GENEROSITY as INFANTILISM is to…, for MATURITY). Most of the relationships between the pairs of terms (A to B and C to D) were based on synonymy, antonymy, part to whole, cause and effect, degree, exemplar- category and object-action relations. Analogy problems were constructed taking into account associative strengths (forward and backward associative strength < .20) according to Spanish free association norms [42,43]. Analogies were chosen from a preliminary normative study in which 57 participants were asked to provide a solution to each problem. The study was conducted to ensure that the experimental items had an appropriate difficulty level. Hence, only those analogies with a success rate ranging from 20% to 80% were selected (for a similar criterion see [17]). The mean percentage of correctly solved analogies was 44.73% (*SD* = 19.75). The nine categories were split into three different sets (BA-DE-MA, CA-PE-FA and DI-RE-FA) to be used in each of the counterbalance conditions as practiced (Rp+ and Rp- items), control (Nrp+ and Nrp- items) or unstudied (Up+ and Up- items) categories. The sets were matched for difficulty level so that there were no reliable differences between them (Group BA-DE-MA mean accuracy = 41.90; Group CA-PE-FA mean accuracy = 48.34; Group DI- RE-TA mean accuracy = 45.02; *p* > .05).

#### Procedure

Participants were randomly assigned to one of the six counterbalanced conditions and were tested individually. They were told that they would participate in two different and separate experiments; one concerning memory and the other related to analogical thinking. Hence, there was not an explicit link between the studied words and the analogy problems. The experimental session went through three main phases: study, retrieval practice and analogical problems test.

#### Study phase

In this first phase, participants were asked to memorize, for an upcoming memory test, word pairs composed of a lexical category represented by the two first letters (syllable) of a set of words and a word that belonged to that category (e.g., BA-Balanza). They were told to pay special attention to the first syllable of the word that identified the category to which the word belonged because this category would be used as a retrieval cue in the upcoming memory test. Each pair was presented in the center of the screen for 5 s with a 1 s inter-stimulus interval. Four pairs were used as fillers and appeared at the beginning and at the end of the list to reduce primacy and recency effects. Thirty-six experimental pairs (6 out of the 9 possible categories) plus the filler ones were presented twice with each pair in each list presented in random order.

#### Retrieval practice phase

In this phase, participants were asked to repeatedly recall words of the previous phase. In each trial, a fixation cross was presented followed by the category label (e.g., CA) for 2 s and then the first three letters of the target word (e.g., Car_____) for 6 s. Participants were asked to recall aloud the studied word which matched with the cue. Only half of the items from half of the studied categories were presented during selective retrieval, which makes a total of nine Rp+ items. They were presented in separate blocks of three words with a filler item at the beginning and the end of each block. The blocks were displayed five times in a pseudorandom order. At the end of this phase, participants completed a distractor task for 5 minutes (completion of basic arithmetical operations; e.g., 3 x 2 + 6).

#### Analogical thinking phase

At the end of the session, participants were instructed to solve analogy problems by finding the relationship between A and B, and by thinking of a word that was related to C in the same way. No reference was made to the previously studied materials and participants were engaged in this phase as part of a different experiment.

They were first given examples of how to solve analogy problems and then provided with two practice problems with solutions. Then, the analogical test started. On each trial, the analogy was presented in the center of the computer screen for a maximum of one minute. Participants were asked to come up with a solution aloud and press the space bar afterward. A total of 54 analogical problems were presented. 36 analogies related to the words studied in the first phase. Eighteen additional problems were not related to any of the studied words and were added as fillers that represented problems with unprimed solutions. Analogies were presented randomly in two separate blocks in order to control for output order effects. First, a block including problems whose possible solutions were related to unpracticed, unrelated unpracticed (control) and unstudied words (Rp-, Nrp- and Up-, respectively) was presented. Then, a block with problems for which the solution word corresponded with practiced, control and unstudied words (Rp+, Nrp+ and Up+) was presented. Each analogy was scored with either 1 (correct) or 0 (incorrect or unsolved) by using a two different scoring procedures: namely, a strict scoring criterion (the response to each analogy was considered correct only if it exactly matched the target word on the study list) to minimize bias during scoring, and a lenient criterion (the response was considered correct as long as it was similar (i.e., a synonym) of the exact word they studied at the first phase).

Finally, participants were given a questionnaire to learn about the strategies they used during each phase (study, retrieval practice and analogical problem solving) and whether they were aware of the relationship between the memory task and the analogy problems. The entire experimental session lasted approximately one hour, depending on the participants’ speed to solve the problems. Presentation of the items in the experiment was controlled by E-prime 2.0 [44].

### Results and discussion

**Fig 1:**
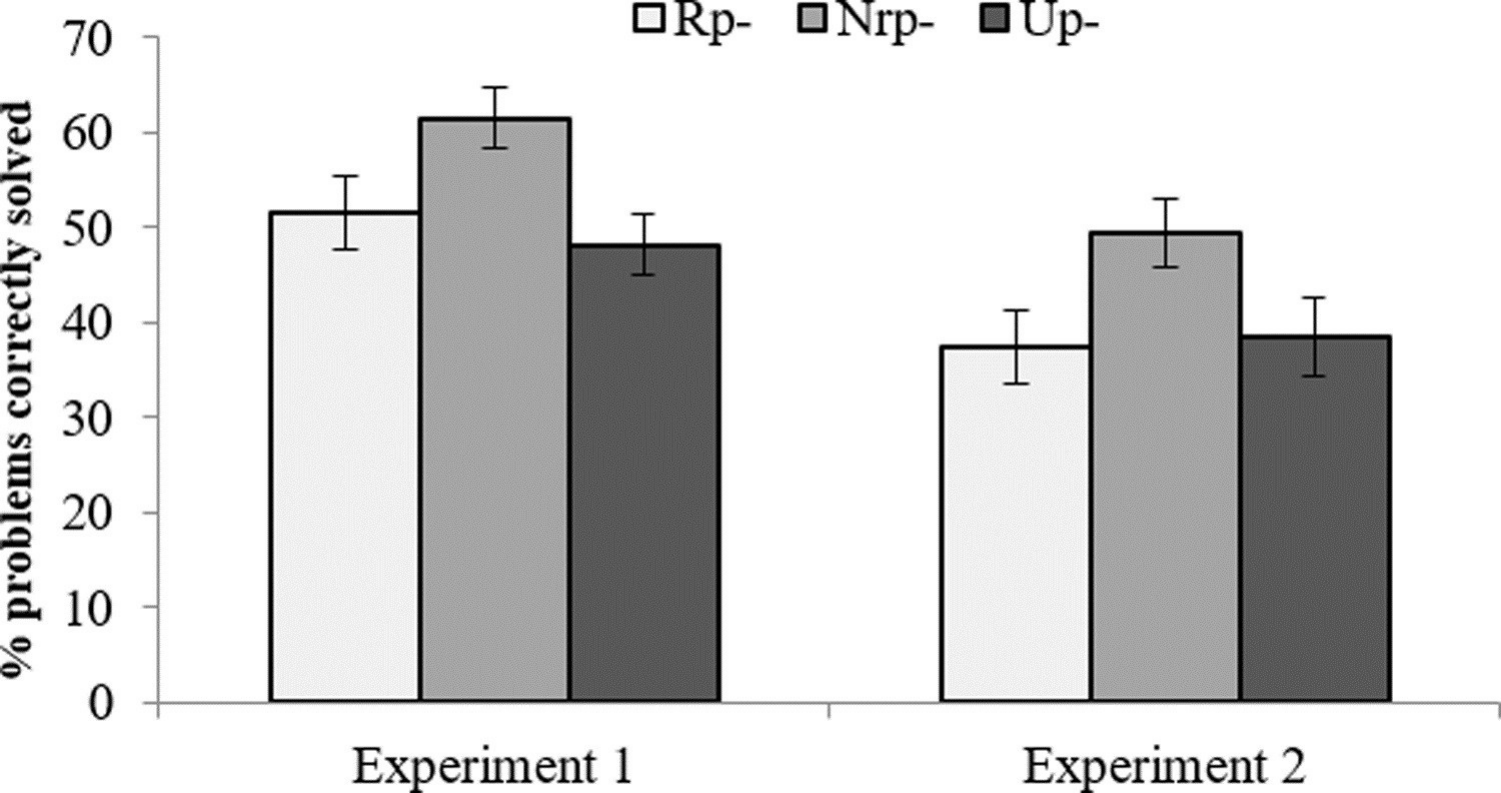
Performance on the analogical test as a function of the status of the solutions (error bars represent the standard error of the mean). Rp- = Solutions that were competitors during retrieval practice. Nrp- = Solutions that were not competitors during retrieval practice. Up- = Solutions that were neither competitors during retrieval practice nor previously studied.

The mean percentage of success during the retrieval practice phase was 61.11 (*SD* = 25.06) and the mean percentage of analogies that were correctly solved was 54.32% (*SD* = 10.09), which reflect a relatively good general performance on the tasks. Fig 1 shows mean percentages of correctly solved analogies and solution times for each type of item. Two separate repeated-measures analyses of variance (ANOVA) were performed on the percentage of analogy problems correctly solved with studied items and on reaction times.

#### Retrieval-induced impairment effect

To assess the negative effect of retrieval practice on analogical problem solving we conducted an ANOVA with type of item (Rp-, Nrp- and Up-) as the factor and accuracy rates as the dependent variable. The analysis applying a strict scoring criteria showed a reliable effect of type of item F(2, 58) = 5.118, MSE = 1444.44, p = .009, η_p_^2^ = .15. Follow-up comparisons revealed that participants solved significantly fewer analogies with Rp- words (*M* = 51.48, *SD* = 21.14) than with Nrp- items (*M* = 61.48, *SD* = 18.16), *t*(29) = -2.162, *p* = .039, *d* = 0.51, indicating that unpracticed words that were related to practiced items were less generated as solutions than unpracticed control items. In addition, Nrp- items (*M* = 61.48, *SD* = 18.16) were reliably more generated as solutions than Up- items (*M* = 48.15, *SD* = 18.30), *t*(29) = 3.19, *p* < .01, *d* = 0.73, which reveals a priming effect in analogical problem solving. Finally, there was no reliable difference between Rp- (*M* = 51.48, *SD* = 21.14) and Up- items (*M* = 48.15, *SD* = 18.30) (*t*(29) < 1, *p* = .43, *d* = 0.16). Hence, the retrieval-induced impairment on studied but unpracticed items was comparable to the effect of not presenting these items for study.

The analysis performed when applying a lenient criterion to score the solutions revealed exactly the same pattern. Thus, there was a reliable effect of type of item F(2, 58) = 6.25, MSE = 1400.55, p = .003, ηp2 = .177. T-tests confirmed that analogies were still solved less frequently with Rp- items (*M* = 58.15, *SD* = 20.57) than with Nrp items (*M* = 67.41, *SD* = 17.73), t(29) = -2.22, p = .034, d = 0.48. In addition, Nrp- items (*M* = 67.41, *SD* = 17.73) were reliably more generated as solutions than Up- items (*M* = 54.07, *SD* = 16.95), t(29) = 3.67, p = .001, d = 0.77, and there was no reliable difference between Rp- (*M* = 58.15, *SD* = 20.57) and Up- items (*M* = 54.07, *SD* = 16.95), t(29) = 1.076, p = .291, d = 0.22.

A similar ANOVA on reaction times (in ms) when applying the strict scoring criteria failed to reveal differences between Rp- (*M* = 7683.93, *SD* = 3932.69), Nrp- (*M* = 8332.64, *SD* = 3830.08) and Up- items (*M* = 8125.51, *SD* = 3871.46), F(2, 58) < 1, MSE = 3293621.58, p = .52, η_p_^2^ = .022. We also failed to observe a reliable effect on reaction times when using a lenient scoring criteria [Rp-: *M* = 8236.70, *SD* = 3530.74; Nrp-: *M* = 8413.03, *SD* = 4119.79; Up-: *M* = 8348.24, *SD* = 3796.31, F(2, 58) < 1, MSE = 238635.65, p = .94, η_p_^2^ = .002].

#### Facilitation effect

To evaluate the possible benefit of retrieval practice on analogical thinking we conducted an ANOVA on accuracy with type of item (Rp+, Nrp+ and Up+ words) as the repeated-measure factor. The analysis using the strict scoring procedure revealed a reliable effect, F(2, 58) = 7.14, MSE = 2392.31, p < .01, η_p_^2^ = .198. Follow-up analyses failed to reveal reliable differences between Rp+ (*M* = 63.33, *SD* = 20.03) and unpracticed (Nrp+) control items [*M* = 55.92, *SD* = 18.33), *t*(29) = 1.55, *p* = .13, *d* = 0.39], even though there was a trend towards better performance for practiced items. We however found a facilitation (priming) effect that resulted from presenting the potential solutions during the first phase of the experiment since Nrp+ problems (those whose solutions were presented during study but belonged to unpracticed categories) were better solved (*M* = 55.93, *SD* = 18.33) than the Up+ problems (*M* = 45.56, *SD* = 15.54), whose solutions were never presented, *t*(29) = 2.11, *p* = .04, *d* = 0.61. In addition, participants significantly produced more Rp+ solutions (*M* = 63.33, *SD* = 20.03) than Up+ solutions (*M* = 45.56, *SD* = 15.54), *t*(29) = 3.97, *p* < .01, *d* = 0.99. The ANOVA on accuracy when using the lenient scoring procedure the effect of type of item did not reach significance, although it was close to it [F(2, 58) = 2.83, MSE = 882.03, p = .07, η_p_^2^ = .089; Rp+: *M* = 66.67, *SD* = 21.04; Nrp+: *M* = 62.59, *SD* = 15.02; Up+: *M* = 55.93, *SD* = 16.11].

The ANOVA performed on reaction times data failed to show a reliable effect of type of item either when using strict [Rp+: *M* = 7807.93, *SD* = 3736.11; Nrp+: *M* = 6486.90, *SD* = 2146.71; Up+; *M* = 7754.53, *SD* = 3446.54; F(2, 58) = 2.86, MSE = 16774321.83, p = .065, η_p_^2^ = .090] or lenient scoring criteria [Rp+: *M* = 8027.45, *SD* = 3731.60; Nrp+: *M* = 6891.41, *SD* = 2319.15; Up+: *M* = 8113.68, *SD* = 3036.54, F(2, 58) = 2.43, MSE = 13959872.96, p = .10, η_p_^2^ = .077].

In an attempt to better understand performance on the analogy task, we looked whether success at retrieval practice predicted either overall accuracy at test or impairment for Rp- items. Pearson correlation analyses failed to showed linear relationships both using the strict (*r* = .250, *p* = .183 and *r* = -.117, *p* = .537, respectively) and the lenient scoring procedures (*r* = .111, *p* = .560 and *r* = -.168, *p* = .376, respectively). This lack of relationship between retrieval practice success and final test accuracy is not surprising since literature on RIF has systematically shown this to be the case (45, 46). Finally, participants’ responses to the questionnaire indicated that 23 out of the 30 participants (77%) became aware of the connection between the memory and the problem-solving tasks and recognized that they tried to solve the analogical problems by recalling the previously studied words. Critically, almost all these participants (73.33%) reported they were aware of such a relation at the end of the final test, when practiced (Rp+) items were presented and they noticed these words could be used as solutions. Hence, it seems reasonable to claim that, at least to some extent, performance during the first block of problems (whose potential solutions were unpracticed and unstudied items) was scarcely modulated by conscious retrieval of studied items. Although some studies have reported reliable RIF effects on response times using recognition memory tests [45], we did not observe any facilitation/inhibition effects on this measure. Given that our analogy test would require a number of processes in addition to recognition (such as mapping and evaluation) response latencies might not be sensitive to the underlying memory control processes involved during analogical reasoning.

In summary, we found that items that competed for retrieval -and presumably were the target of inhibitory control during the practice phase- were selected to a lesser extent as solutions of the analogical problems than control items. This was so in the context of a separate problem-solving task that most participants failed to connect with the previous stages of the experimental session. Hence, this finding joins previous results to show that the cost of accessibility that follows selective retrieval may also be observed on tasks requiring more than only recalling specific episodic memories. Thus, in addition to the impact that memory inhibition has shown on decision making [33] and the resolution of creativity problems [34], the present results indicate that retrieval-induced forgetting may also hamper analogical reasoning.

An additional contribution of the present experiment is that it provides a complementary measure of the disruptive effect that memory inhibition may have on the accessibility to potential solutions on analogical problem solving. Specifically, here we were able to more precisely estimate the selective impairment for inhibited solutions by including problems whose potential solutions were never presented in the context of the experiment. Hence, Up- solutions provide a baseline measure of the effect of the previous presentation (study) of the items that could become solutions. Given that the generation of Rp- solutions during analogical thinking was similar to that for Up- solutions, we interpret that inhibitory control during selective retrieval lowered the accessibility of competing memories so that participants behaved as if Rp- solutions had not been previously presented.

## Experiment 2

The results of Experiment 1 indicated that reduced access to previously inhibited information influenced analogical problem solving by reducing the probability of coming up with a solution if it was previously inhibited during selective retrieval. In designing Experiment 1, we assumed that the access to targets to solve analogies might be implicit, since we made a serious effort through instructions to separate the memory and analogy tasks by making participants believe that these tasks were completely independent of each other. Despite this, most participants reported that they ended up being aware of the possibility of using the studied words to solve the problems. Hence, it could be the case that rather than testing whether inhibition of relevant information could implicitly affect performance on the analogical test, we were directly assessing episodic memory. That is, during problem solving participants could have attempted to think back on the words previously studied in order to solve analogies without genuinely applying analogical mapping processes. This would especially be so for the Rp+ critical items, which were repeatedly presented during the practice phase of the experiment. Interestingly, in our procedure the analogies that could be solved with practiced (Rp+) and unpracticed (Rp- and Nrp-) items were presented in two different blocks. Thus, participants first attempted to solve Rp- and Nrp- analogies and then moved to the block with analogies whose solutions could be Rp+ and Nrp+ items. Therefore, those who reported awareness of the relationship between the two tasks at the half or the end of the problem-solving test might have noticed the connection while they were solving the second block of analogies. If so, they might have thought back only during the second testing block. In order to better determine to what extent participants implicitly accessed potential solutions to solve the analogies without explicitly using episodic retrieval, we conducted a second experiment where the procedure for the study and practice phases was identical to that used in Experiment 1, but the analogical test only included analogies that could be solved with Rp-, Nrp- and Up- items. Hence, participants were not given problems whose potential solutions were Rp+, Nrp+ and Up+ items. We expected this testing procedure to minimize the participants’ awareness of the relationship between the memory and the reasoning stages of the experimental session. The new procedure would allow us to replicate the main finding of Experiment 1 as well as to precise to what extent participants may deal with analogy problems without noticing the above-mentioned relationship.

### Method

#### Participants

Based on the results obtained in Experiment 1, and before starting to conduct Experiment 2, we decided to have the same sample size in the present experiment. Thus, thirty undergraduate students (mean age = 19.87 years; *SD* = 1.45) from the University of Granada participated in the experiment for course credit. None of them had participated in the previous study. All participants were native Spanish speaker, had normal or corrected-to-normal vision and gave their written consent to participate in the experiment by signing the appropriate informed consent paperwork. The Ethics Committee of the University of Granada approved the procedure of this study.

#### Material and procedure

The material and procedure were the same as in Experiment 1, except that in the final (problem solving) test participants only solved analogies whose solutions corresponded to Rp-, Nrp- and Up- items.

### Results and discussion

Only 8 (27% of the) participants reported being aware of the relation between the memory and the analogy tasks, which indicates that the present procedure was successful (in comparison to that of Experiment 1; two samples proportion test with *p* < 0.01) at enhancing the implicit nature of the analogical test. These eight participants exhibited a totally different pattern of performance on the problem-solving test in comparison to the remaining participants. Specifically, they produced more Rp- (M = 73.61, SD = 10.18) than Nrp- solutions (M = 59.72, SD = 10.18) to the problems, even though a Wilcoxon test failed to reveal a reliable effect. Hence, eliminating problems related to Rp+ items from the analogical test drastically reduced awareness of the relation between the different stages of the experiment. Since we wanted to assure that the results were not due to explicit memory strategies, the data from the eight participants who noticed the relationship between the two tasks were removed from the analyses. It is worth mentioning that these participants explicitly indicated that they attempted to recall words from the memory task to solve analogies.

The mean percentage of recall at the retrieval practice phase was 70.20 (*SD* = 22.32), while the mean percentage of correctly solved problems was 39.31 (*SD* = 13.88). Neither accuracy rates during analogical reasoning nor RIF scores correlated with retrieval practice success (*r* = .127, *p* = .574; *r* = -.290, *p* = .190, respectively). Fig 1 shows the mean percentages of correctly solved analogies and solution times as a function of the type of solution (Rp-, Nrp- and Up-).

Retrieval-induced impairment effect. A repeated-measures ANOVA on accuracy using the strict scoring procedure showed a reliable effect of type of item (Rp-, Nrp- and Up-), F(2, 42) = 4.89, MSE = 995.14, p = .01, η_p_^2^ = .189. T-tests confirmed that participants came up with fewer Rp- items (*M* = 37.37, *SD* = 18.32) than Nrp- items (*M* = 49.49, *SD* = 16.7) to solve analogies, *t*(21) = -2.767, *p* = .012, *d* = 0.69. Nrp- solutions (*M* = 49.49, *SD* = 16.7) were more produced than Up- solutions (*M* = 38.38, *SD* = 19.31), *t*(21) = 2.499, *p* = .021, *d* = 0.62, which again demonstrates the effect of exposing potential solutions to participants. Also like in Experiment 1, no statistical difference was observed between Rp- (*M* = 37.37, *SD* = 18.32) and Up- solutions (*M* = 38.38, *SD* = 19.31), *t*(21) = -.249, *p* = .806, *d* = .05, indicating that even though Rp- items were presented at study, they behaved as if they had never been presented in the context of the experiment. The ANOVA performed on accuracy when applying the lenient scoring criterion also showed a reliable effect of item type, F(2, 42) = 9.14, MSE = 1060.60, p = .001, η_p_^2^ = .303. Follow-up analysis showed that participants generated fewer Rp- items (*M* = 42.93, *SD* = 16.19) as solutions to the analogies compared to Nrp- items (*M* = 56.57, *SD* = 14.10), *t*(21) = -4.29, *p* = .000, *d* = 0.90. In addition, Nrp- items (*M* = 56.57, *SD* = 14.10) were reliably more generated as solutions than Up- items (*M* = 47.47, *SD* = 17.88), *t*(21) = 3.15, *p* < .005, *d* = 0.57, which again reveals a priming effect in analogical problem solving. Finally, there was no reliable difference between Rp- (*M* = 42.93, *SD* = 16.19) and Up- items (*M* = 47.47, *SD* = 17.88) (*t*(21) = -1.25, *p* = .22, *d* = 0.26). Hence, the retrieval-induced impairment on studied but unpracticed items was comparable to the effect of not presenting these items for study.

The ANOVA performed on reaction times failed to show a significant effect [F(2, 42) = 1.19, MSE = 11552788.20, p = .165, η_p_^2^ = .082; Rp-: *M* = 6773.50, *SD* = 3144.04; Nrp-: *M* = 8189.96, *SD* = 1950.71; Up-: *M* = 7216.01, *SD* = 3849.35]. The same null effect emerged when using the lenient scoring criteria [Rp-: *M* = 8633.51, *SD* = 2986.51; Nrp-: *M* = 9515.41, *SD* = 4004.48; Up-: *M* = 9130.52, *SD* = 3056.44, F(2, 42) < 1, MSE = 1748769.37, p = .44, η_p_^2^ = .038].

In summary, and replicating Experiment 1, Experiment 2 reveals that items that putatively had been the target of inhibitory control were significantly less chosen as solutions than control items in an independent analogical test. Moreover, inhibited words were produced to the same degree than unstudied (unprimed) words. Since in the present experiment additional measures were taken to minimize participants’ awareness about the connection between the memory and the reasoning tasks, these results suggest that the modulation of the accessibility by means of inhibitory mechanisms may impair analogical thinking implicitly.

## General discussion

Current evidence suggests that prior exposure to relevant information can foster analogical problem solving by increasing the accessibility to appropriate knowledge [9,17,21]. In the present work, we aimed to further explore the relationship between memory and analogical thinking from a different angle; namely, that reduced access to critical information may adversely affect performance on an analogical thinking task. The results of two experiments support this idea.

Capitalizing on an experimental procedure (retrieval practice) that has systematically shown to be effective to reduce memory accessibility (for a meta-analytic review see [46]), we conducted two experiments wherein participants engaged in solving analogical problems of the type ‘A is to B as C is to D’ after selectively retrieving part of previously encoded items. Since selective retrieval is known to lead to forgetting (a direct measure of reduced accessibility) of memories that are related to those selectively recalled, we expected selective retrieval to negatively modulate analogical thinking performance provided that the forgotten information turned out to be relevant during problem solving. In this line, we systematically found that related unpracticed (Rp-) words were less chosen as solutions than unrelated control (Nrp-) words to solve analogies. To put it another way, the analogy problems that could potentially be solved with the less accessible items turned out to be more difficult to solve. Importantly, this was the case in two experiments even when participants were not aware that some of the solutions that they generated to solve the problems had been previously presented. Hence, the present results are consistent with others from previous studies that have examined the influence of memory control processes over performance in tests of verbal creativity [34], or decision making [33,47] (for related studies see the meta-analytic review by [47]). In fact, [34] reported similar results regarding how reduced access to relevant information may impair creative thinking. In a series of experiments, participants studied a set of words and then repeatedly practiced a subset of them. Finally, participants were told to solve problems from a Remote Associates Test (RAT). The RAT is a creativity verbal test in which solvers are presented with three unrelated words and they are asked to find a fourth word that is associated with the three unrelated presented words (e.g., for fish-mine-rush, a correct solution could be gold). Words that had previously been the putative target of inhibitory control (by virtue of selective retrieval) were generated to a lesser extent than creative solutions relative to baseline items. Therefore, all these studies are informative of how reduced accessibility to relevant information may impact on thinking, with the present experiment demonstrating for the first time that this also applies to analogical reasoning.

As previously mentioned, a remarkable point of the present experiments is that they were designed to keep participants’ awareness of episodic retrieval to a minimum during problem solving. Analogical thinking has often been viewed as an intentional analytic process [16]. People must deliberately search through potential, and often irrelevant, solutions in memory that can be implemented to the current situation. As mentioned, some have argued that the presence of explicit cues are required to apply previously activated potential solutions to analogical problem solving for successful transfer [23,24]. However some studies also suggest analogical transfer could also occur without explicit cues and without awareness that potential solution was previously presented [2,9,17,20–22]. Along this line, the main finding of the present experiments indicates that information involved in a memory task may later influence how accurately people deal with analogical problems and, what is of special relevance here, do so unwittingly. In Experiment 1 almost all the participants reported being aware that some of the solutions they produced were words that had previously appeared in the experimental session. However, most of them noticed the relation between the two sessions at the half/end of the test when practiced words (Rp+, and their controls Nrp+ and Up+) were presented. Hence, it seems reasonable to think that solutions produced during the first block (containing the most relevant items here: Rp-, Nrp- and Up-) stemmed from genuine analogical mapping processes rather than from retrieval strategies. Experiment 2, which was conducted after removing the second block at test, confirmed this idea by showing that only 26% of the participants became aware of the studied words to solve problems. This experiment again revealed that putatively inhibited (Rp-) solutions were less produced than control (Nrp-) solutions, even when participants who were aware of the relation between the two experimental sessions were not considered. This finding supports the idea that memory can influence reasoning unconsciously. People may attempt to access potential solutions without being aware that their particular memory state (determined by previous retrieval dynamics) may be guiding the current process of problem solving. As a consequence, reasoning may be disrupted by a temporary reduced access to potential solutions in memory.

While a few situational factors might potentially reduce accessibility to relevant information during problem solving (e.g., short encoding time, blocking by other more salient information), the impairment in analogical problem-solving observed in the present experiments may be understood as an aftereffect of inhibitory control during selective retrieval [26,30,32]. By this view, inhibition is an adaptive control mechanism that temporarily downregulates competing memory representations to overcome retrieval interference over target memories, with a wealth of data from behavioral (e.g., [30,48,49]) and brain-related studies supporting this view (e.g., [50–54]). Hence, if the suppressed information turns out to be later relevant and access to it is required, impairment in the ability to come up with such information is to be expected, regardless of the situation requiring its use.

Although an interference-based account of retrieval-induced performance impairment have been proposed in the context of episodic memory testing [55,56], the main present finding represents a serious challenge for such a view (see also [30,33]). Thus, for example, it has been proposed that associative blocking rather than inhibition could be the mechanism that underlies such impairments [56,57]. The idea is that because retrieval practice strengthens the relation between the retrieval cue and the practiced (Rp+) items relative to the strength of the unpracticed (Rp- and Nrp-) items, if the cue is presented in a later test, practiced items’ memories would have a greater probability of being activated and will compete with their related (Rp-) memories so as to block their retrieval. This account, however, is not able to accommodate findings of retrieval-induced performance impairments observed with testing procedures that do not, either overtly or covertly, allow for the use of the memory cues provided during the retrieval practice phase (see [30], for a discussion of this issue). As a matter of fact, the separate analyses of the performance of the (eight) participants who reported being aware that the studied/practiced words could be used to solve the problems revealed that thinking back on these words tended to enhance rather than hinder performance (for related results see [30]). Hence, since in our experiments (Experiment 2 in particular) participants were tested with analogical problems and presented with items that were totally unrelated to the practiced items, it would seem odd to claim that blocking from the more recently processed (Rp+) items was taking place during the contextually unrelated analogical thinking phase. The implicit memory nature of the testing procedure of Experiment 2 supports further the interpretation that it was memory inhibition what hindered analogical problem solving (see [34] for a similar interpretation applied to verbal creativity). Moreover, the inclusion in the final test of analogies whose possible solutions were items that had never been presented in the context of the experiment enables us to precise the degree of inaccessibility that selective retrieval may cause on problem solving. Since participants came up with suppressed (Rp-) solutions as little as they did with unstudied (unprimed; Up-) solutions, it seems that executive control at retrieval may downregulate competing memories by rendering them as if they had not been recently encoded. Thus, while it does not seem to be the case that problem solving may be generally affected by selective retrieval (Nrp+ solutions were more produced than unprimed Up- solutions), it seems reasonable to put forward that inhibitory control during retrieval may lessen accessibility of some potential solutions.

Of course, at first sight, one might have expected generation of Rp- solutions to be even lower than that of Up- solutions because of inhibitory control. However, it should be noticed that Rp- items differ from Up- items in that they were previously presented in the context of the experiment during the study phase (in addition to being the target of inhibitory control). Hence, Rp- items would enjoy some benefit from this previous exposure (a ‘priming’ effect). If so, inhibition would be expected to take from them this benefit reducing their accessibility to the level of non-exposed items. Future experiments where perceptual and lexical priming from previous exposure is avoided should be conducted to further support this interpretation.

An additional point that deserves attention is that we observed the same pattern of retrieval-induced impairment with different (strict vs. lenient) scoring procedures, which is suggestive of how inhibitory control worked during selective retrieval practice. Because the tolerant criterion involved accepting as correct items that did not exactly match the studied items (i.e., synonymous or semantically related words), the fact that participants also solved less Rp- than Nrp- analogies when considering this less strict criterion suggests that inhibitory control during selective retrieval not only affects episodic memory, but also may affect general semantic representations (for a similar idea on a related memory inhibition phenomenon (see [58]).

While our main finding unveils the negatives consequences of memory control mechanisms on problem-solving solutions accessibility, previous work has already shown that inhibitory control recruited during problem solving may also have the potential for facilitating performance by preventing interference of irrelevant information. Thus, for instance, in the context of creative problem solving, Storm, Angello, and Bjork [59] used a problem-solving-induced forgetting paradigm in which participants were exposed to cue-response pairs (e.g., lick–tongue, sprinkle–rain) and then were asked to solve RAT problems. Critically, half of the cue-response pairs contained misleading associates for the RAT problems designed to interfere and cause fixation (i.e., lick, sprinkle, mines) whereas half of the RAT problems did not (i.e., manners, tennis, round). Performance in a final cue-response test showed that participants recalled fewer response words associated with cues that appeared during the problem- solving phase than response words associated with cues that did not appear during the problem-solving phase. Thus, attempting to solve a problem caused participants to forget irrelevant information in order to deal with interference from competing misleading associates. Similarly, metaphor processing may share similar features with problem solving suggesting a link between overcoming interfering irrelevant information and inhibitory control. George and Wiley [60] conducted a series of experiments using a metaphor-induced lexical forgetting paradigm, in which participants studied word pairs composed on potentially metaphoric vehicles cues and literal associate targets (e.g. SHARK–swim). Then, participants read and interpreted half of the vehicles as part of metaphoric sentences (e.g. The lawyer for the defense is a shark). On a test of final recall for all of the initially studied word pairs, participants showed reduced recall for word pairs consisting of vehicles and their literal associates. These results suggest that, in order to arrive at the figurative meaning of a metaphor, literal irrelevant associated information that is previously activated may have to be inhibited. As a consequence of attempting to retrieve the figurative target meaning from memory, recall for literal information is impaired. Therefore, the relationship between memory control processes, such as inhibition, and problem solving may depend on which specific information is the target of control. If inhibition acts on information in memory that would subsequently have to be retrieved to generate a solution, worse problem solving performance would be expected. On the contrary, if inhibition is recruited to suppress activation of irrelevant information that would otherwise interfere with problem solving, one could predict enhanced performance.

Our main findings might have applied implications, since analogical thinking is thought to be crucial in a variety of fields ranging from scientific discovery to creative problem solving. Despite the fact that analogical reasoning is a desirable tool to tackle real-world problems, a number of studies have shown that people often fail to use past knowledge to solve a problem which shares similar features [4,5,25,61]. Since performance can be influenced by how available certain pieces of information are in our memory, every single thought or mental activity done immediately before solving a problem might modulate a particular memory state. In fact, our study suggests that the consequences of certain cognitive operations over memory representations may hamper analogical reasoning without noticing it. Although successful retrieval can facilitate the recall of wanted memories, it can also impair later access to related memories. In a more naturalistic setting, merely attempting to generate analogical solutions to a problem by discussing with other people on previous experiences could prevent us from solving the problem. For example, in a brainstorming session, wherein the main goal is to generate as many different ideas as possible, overhearing or coming up with some ideas or solutions about a particular problem might lead to inhibition of potential related solutions or ideas [62]. To conclude, the modulation of the accessibility of information by means of an inhibitory control mechanism may have significant effects on analogical reasoning. Our study adds to a growing body of research to show that unconscious control processes related to retrieval may have an impact on high-order cognitive operations such as creative thinking [34] or decision-making [33,47]. Future studies on this topic are required in order to understand the extent to which unconscious processes may influence our complex behavior.

## Supporting information

S1 Table Supplementary materials. Orthography-Based Word Categories and analogical problems used in Experiments 1 and 2.
(PDF)
