# Supplementary material for: Inhibitory control during selective retrieval may hinder subsequent analogical thinking
Source: PLoS One. 2019 Feb 12;14(2):e0211881. doi: 10.1371/journal.pone.0211881 (PMC6372166; doi:10.1371/journal.pone.0211881)
Supplement: S1 Table — Orthography-Based Word Categories and analogical problems used in Experiments 1 and 2. (PDF) [file pone.0211881.s001.pdf]

S1 Table.

Practiced Status	BA	Analogy	MA	Analogy
Rp+/Nrp+/Np+ items	Bambú	ARDILLA es a BELLOTA como OSO PANDA es a…	Maquillaje	LIBRO es a BIBLIOTECA como COLORETE es a...
	Balanza	PAZ es a PALOMA como JUSTICIA es a…	Marinero	PARQUE es a NIÑOS como BARCO es a…
	Bañera	DORMIRSE es a CAMA como LAVARSE es a…	Matanza	LIEBRE es a CACERÍA como CERDO es a…
Rp-/Nrp-/Np- items	Bandera	GOLPEAR es a MARTILLO como IZAR es a…	Madurez	AVARICIA es a GENEROSIDAD como INFANTILISMO a...
	Basura	DISPUTA es a PELEA como DESPERDICIOS es a…	Maleta	ENCICLOPEDIA es a LIBRO como EQUIPAJE es a…
	Batalla	BERLÍN es a MURO como TRAFALGAR es a…	Manual	DIVERSIÓN es a ABURRIMIENTO como AUTOMÁTICO es a…

Practiced Status	DE	Analogy	CA	Analogy
Rp+/Nrp+/Np+ items	Detective	BISTURÍ es a CIRUJANO como LUPA es a…	Canario	PASTOR es a ALEMÁN como PLÁTANO es a...
	Delito	HEPATITIS es a ENFERMEDAD como HOMICIDIO es a…	Capitán	CIUDAD es a ALCALDE como BARCO es a…
	Debate	ARMA es a GUERRA como ARGUMENTO es a...	Caracol	CABALLO es a CEBRA como BABOSA a…
Rp-/Nrp-/Np- items	Desastre	IRA es a FURIA como CATÁSTROFE es a…	Cabello	CIELO es a TOCINO como ÁNGEL es a…
	Deporte	PASTEL es a REPOSTERÍA como ATLETISMO es a…	Camarero	MUEBLE es a CARPINTERO como CÓCTEL es a…
	Democracia	LIBERTAD es a ESCLAVITUD como DICTADURA es a…	Catedral	PENSIÓN es a HOTEL como IGLESIA es a…

Practiced Status	PE	Analogy	FA	Analogy
Rp+/Nrp+/Np+ items	Pedazo	ESTUDIANTE es a ALUMNO como TROZO es a...	Fauna	ESTRELLA es a CONSTELACIÓN como ANIMAL es a...
	Pesimismo	ALEGRÍA es a ENTUSIASMO como NEGATIVIDAD a…	Fallo	SUEÑO es a REALIDAD como ACIERTO es a...
	Petición	PROHIBICIÓN es a NEGACIÓN como SOLICITUD es a...	Farmacia	FRUTA es a MERCADO como MEDICAMENTO es a…
Rp-/Nrp-/Np- items	Pelota	CUBO es a DADO como ESFERA es a...	Fábrica	HOSTELERÍA es a RESTAURANTE como INDUSTRIA es a…
	Península	BALEAR es a ARCHIPIÉLAGO como IBÉRICA es a…	Fantasía	RISA es a COMEDIA como DRAGÓN es a…
	Pereza	GLOTONERÍA es a GULA como VAGUEZA es a...	Famoso	ROBUSTO es a GORDO como CÉLEBRE es a…

Practiced Status	DI	Analogy	RE	Analogy
Rp+/Nrp+/Np+ items	Digestión	MECÁNICO es a REPARACIÓN como ESTÓMAGO es a…	Rebaño	PECES es a BANCO como OVEJAS es a…
	Dilema	DESEO es a ANHELO como PROBLEMA es a...	Receta	LAVADORA es a INTRUCCIONES como COMIDA es a…
	Divorcio	ALIVIO es a ANGUSTIA como MATRIMONIO es a…	Relámpago	NUBLADO es a LLUVIA como TRUENO es a...
Rp-/Nrp-/Np- items	Diciembre	SEMANA es a DOMINGO como AÑO es a...	Regalo	FATIGA es a CANSANCIO como OBSEQUIO es a…
	Difunto	REY es a MONARCA como FALLECIDO es a...	Restaurante	CATÁLOGO es a SUPERMERCADO como MENÚ es a…
	Diseño	CANCIÓN es a COMPOSICIÓN como ROPA a…	Retrato	CAMA es a LECHO como FOTOGRAFÍA es a...

Practiced Status	TA	Analogy
Rp+/Nrp+/Np+ items	Tango	BRASIL es a SAMBA como ARGENTINA es a...
	Tarjeta	TRÁFICO es a MULTA como FÚTBOL es a…
	Tacto	SONIDO es a OÍDO como CARICIA es a…
Rp-/Nrp-/Np- items	Taller	FLOR es a CAMPO como HERRAMIENTA es a…
	Taza	FLOR es JARRÓN como CAFÉ es a…
	Tabaco	PROTEÍNA es a POLLO como NICOTINA es a…
